# Efficient fermentative production of polymer-grade d-lactate by an engineered alkaliphilic *Bacillus* sp. strain under non-sterile conditions

**DOI:** 10.1186/s12934-015-0408-0

**Published:** 2016-01-12

**Authors:** Nilnate Assavasirijinda, Deyong Ge, Bo Yu, Yanfen Xue, Yanhe Ma

**Affiliations:** State Key Laboratory of Microbial Resources, Institute of Microbiology, Chinese Academy of Sciences, Beijing, 100101 People’s Republic of China; CAS Key Laboratory of Microbial Physiological and Metabolic Engineering, Institute of Microbiology, Chinese Academy of Sciences, Beijing, 100101 People’s Republic of China; University of Chinese Academy of Sciences, Beijing, 100049 People’s Republic of China; Tianjin Institute of Industrial Biotechnology, Chinese Academy of Sciences, Tianjin, 300308 People’s Republic of China

**Keywords:** Alkaliphilic, *Bacillus* sp., d-lactate, Non-sterile fermentation, Peanut meal

## Abstract

**Background:**

Polylactic acid (PLA) is one important chemical building block that is well known as a biodegradable and a biocompatible plastic. The traditional lactate fermentation processes need CaCO_3_ as neutralizer to maintain the desired pH, which results in an amount of insoluble CaSO_4_ waste during the purification process. To overcome such environmental issue, alkaliphilic organisms have the great potential to be used as an organic acid producer under NaOH-neutralizing agent based fermentation. Additionally, high optical purity property in d-lactic acid is now attracting more attention from both scientific and industrial communities because it can improve mechanical properties of PLA by blending l- or d-polymer together. However, the use of low-price nitrogen source for d-lactate fermentation by alkaliphilic organisms combined with NaOH-neutralizing agent based process has not been studied. Therefore, our goal was the demonstrations of newly simplify high-optical-purity d-lactate production by using low-priced peanut meal combined with non-sterile NaOH-neutralizing agent based fermentation.

**Results:**

In this study, we developed a process for high-optical-purity d-lactate production using an engineered alkaliphilic *Bacillus* strain. First, the native l-lactate dehydrogenase gene (*ldh*) was knocked out, and the d-lactate dehydrogenase gene from *Lactobacillus delbrueckii* was introduced to construct a d-lactate producer. The key gene responsible for exopolysaccharide biosynthesis (*epsD*) was subsequently disrupted to increase the yield and simplify the downstream process. Finally, a fed-batch fermentation under non-sterile conditions was conducted using low-priced peanut meal as a nitrogen source and NaOH as a green neutralizer. The d-lactate titer reached 143.99 g/l, with a yield of 96.09 %, an overall productivity of 1.674 g/l/h including with the highest productivity at 16 h of 3.04 g/l/h, which was even higher than that of a sterile fermentation. Moreover, high optical purities (approximately 99.85 %) of d-lactate were obtained under both conditions.

**Conclusions:**

Given the use of a cheap nitrogen source and a non-sterile green fermentation process, this study provides a more valuable and favorable fermentation process for future polymer-grade d-lactate production.

## Background

Polylactic acid is an important chemical building block that is known to be a biodegradable and a biocompatible plastic [1]. Polylactic acid is usually produced from optically pure l-lactic acid. However, the use of l- and d-lactic stereocomplexation (racemic crystallite) has mechanical properties greater than either pure l- or d-polymer [2]. Furthermore, impure l- and d-isomers will form an amorphous polymer that is not satisfactory for industrial applications [3, 4]. Because optical purity is an essential quality of a final product, biological processes have been widely used to produce lactate monomers, as they can produce a single stereoisomer (l- or d-lactic), while chemical syntheses can only produce a racemate [5]. Thus, as a suitable modifier of biodegradable poly l-lactic acid, high-optical-purity d-lactic acid is attracting increasing attention, both in academia and industry. However, few studies have focused on the microbial production of d-lactic acid [6], while l-lactic acid production has been well studied [7, 8].

Notably, lactic acid is listed as one of the top 30 potential building block chemicals produced from biomass; thus, identifying cheap substrate sources and easy handling processes is economically important [8]. Several efforts have been made to use low-priced nitrogen sources to substitute for yeast extract during lactate production. Among these, peanut meal has proven to be the best, as it can promote high-yield lactate production from glucose [9, 10]. Additionally, the use of non-sterile conditions in industrial fermentations would reduce the need for equipment, as well as lower energy consumption and labor costs. These factors can be especially important for low-cost, high-volume chemical lactic acid production [5, 11].

Additionally, traditional lactate fermentation processes require calcium carbonate as a neutralizer to maintain the desired pH, which results in the production of insoluble calcium sulfate waste during the purification process. To overcome this environmental issue, other neutralizers, such as Na^+^ or NH_4_^+^, have been applied in lactate fermentations, although the production titers of current lactic acid producers have not been satisfactory because of the high toxicity of Na^+^ to these strains [12]. In an effort to overcome these limitations, it has been suggested that alkaliphilic organisms have the potential to be used as organic acid producers in a NaOH-neutralizing agent-based fermentation, depending on their monovalent sodium ion tolerance [13]. Moreover, their tolerance to high salt levels and pH could also minimize contamination from other organisms during industrial fermentation [14]. Some reports have verified the great potential of using alkaliphilic organisms for l-lactate production [9, 14, 15]. However, the use of alkaliphiles for d-lactate production has never been reported.

*Bacillus* sp. N16-5 was previously isolated from Wudunur Soda Lake in Inner Mongolia, China, and it is an alkaliphile that exhibits optimum growth at pH 10.0 [16]. It can utilize various types of sugars, such as pentoses, hexoses, and polysaccharides [17, 18]. Importantly, a genetic manipulation system has been successfully developed for this promising strain [19]. Thus, these characteristics make *Bacillus* sp. N16-5 as an ideal host for developing an alkaliphilic d-lactate producer. In this study, the alkaliphilic *Bacillus* sp. N16-5 strain was engineered to become a d-lactate producer. The fermentation procedure was optimized to use cheap peanut meal as a nitrogen source in combination with a non-sterile fermentation process in which NaOH was used as a neutralizing agent. Using these strategies, a high yield, a high level of optical purity, and a high titer of d-lactic acid were obtained in a fed-batch fermentation process.

## Results and discussion

### Exploration of the sodium lactate tolerance of *Bacillus* sp. N16-5

**Fig. 1:**
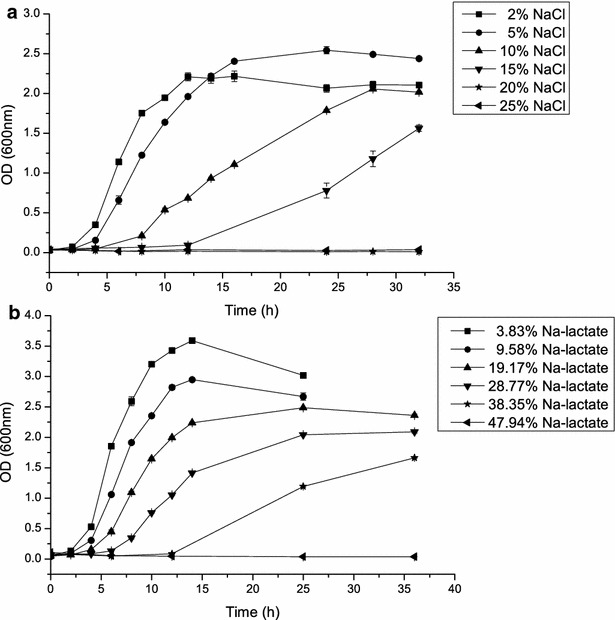
Growth curves of *Bacillus* sp. N16-5 in Horikoshi medium containing sodium chloride or sodium lactate. **a** *Bacillus* sp. N16-5 was cultivated in Horikoshi medium supplemented with different concentrations of NaCl. **b** *Bacillus* sp. N16-5 was cultivated in Horikoshi medium supplemented with different concentrations of sodium lactate. *Error bars* represent the standard deviations of three replicates

The *Bacillus* sp. N16-5 strain is an alkaliphile that tolerates high concentrations (0–15 %, w/v) of NaCl [17]. Wu et al. [20] reported that adapting the *E. coli* ALS1187 strain to high NaCl concentrations significantly improved lactate production. Therefore, it can be expected that a high sodium ion tolerance could improve lactate production. Thus, the sodium lactate tolerance of *Bacillus* sp. N16-5 was determined. To do so, the effects of various concentrations of NaCl and sodium lactate on bacterial growth were investigated. *Bacillus* sp. N16-5 showed the best growth in 5 % NaCl and 3.83 % sodium lactate (equal to 2 % NaCl) (Fig. 1). It should be noted that the growth curve in 5 % NaCl resembled that in 19.17 % sodium lactate (equal to 10 % NaCl based on Na^+^ molar concentration). Thus, the *Bacillus* sp. N16 strain is more tolerant of sodium lactate, which indicates that *Bacillus* sp. N16-5 is an ideal host for lactate production.

### Engineering *Bacillus* sp. N16-5 to become a d-lactate producer

First, a 954-bp fragment of the l-lactate dehydrogenase gene (*L*-*ldh*) was knocked out. Then, the *D*-*ldh* gene, which is responsible for d-lactate formation, from *L. delbrueckii* was expressed under the control of the native *Bacillus* sp. N16-5 *L*-*ldh* promoter in plasmid pMK4. The recombinant vector, named pDlac, was subsequently transformed into the ∆*ldh* strain. The resultant *Bacillus* sp. N16-5∆*ldh*-pDlac strain only produced d-lactate. Then, its growth, glucose utilization, and production of lactic acid and other organic acids were compared to those of the wild-type (WT) parent strain and the ∆*ldh* strain (Table 1). The ∆*ldh* strain grew slightly slower than the WT strain, and it accumulated greater concentrations of byproducts, especially pyruvate, than the WT strain under aerobic or anaerobic conditions. Our results resemble to those of Kabir et al. [21], who also demonstrated that an *E. coli ldhA* mutant grew slightly slower than a WT strain. The *Bacillus* sp. N16-5-∆*ldh* strain showed higher pyruvate accumulations of 5.39 ± 0.19 and 4.35 ± 0.04 g/l in aerobic and anaerobic conditions, respectively, when compared with those of the WT strain. These characteristics may take an advantage to channels more pyruvate to the d-lactate pathway easily in the further engineering step. The introduction of an exogenous d-lactate dehydrogenase gene complemented the ∆*ldh* strain, as the growth and byproduct accumulation of the ∆*ldh*-pDlac strain were similar to those of the WT strain (data not shown). This implies that promoting the d-lactate pathway restores a metabolic flux balance to the WT strain. Moreover, the ∆*ldh*-pDlac strain accumulated higher levels of d-lactate than the level of l-lactate of the WT strain, while its accumulation of acetate was significantly lower than that of the WT strain, which could be due to the higher expression level of the *D*-*ldh* gene compared with that of the native *L*-*ldh* gene in the WT strain. This experiment shows that the ∆*ldh*-pDlac strain is a promising d-lactate producer, and that it accumulates lower concentrations of byproducts.

**Table 1:** Lactate and byproducts accumulations in the wild-type and engineered strains under aerobic and anaerobic conditions

Strain	Pyruvate (g/l)	Succinate (g/l)	Lactate (g/l)	Formate (g/l)	Acetate (g/l)
Aerobic					
WT	0.27 ± 0.05	0.54 ± 0.13	5.39 ± 0.11^a^	0.37 ± 0.11	4.78 ± 0.84
∆*ldh*	5.39 ± 0.19	3.63 ± 0.88	0.24 ± 0.06^a^	0.92 ± 0.15	5.51 ± 1.21
∆*ldh*-pDlac	0.26 ± 0.09	0.32 ± 0.10	6.32 ± 0.19^b^	0.16 ± 0.08	2.95 ± 0.18
Anaerobic					
WT	0.22 ± 0.11	0.50 ± 0.17	5.28 ± 0.10^a^	1.14 ± 0.19	1.34 ± 0.06
∆*ldh*	4.35 ± 0.04	1.09 ± 0.50	0.10 ± 0.00^a^	2.86 ± 0.25	2.07 ± 0.84
∆*ldh*-pDlac	0.18 ± 0.06	0.53 ± 0.18	6.95 ± 0.02^b^	0.54 ± 0.11	1.17 ± 0.32

WT, *Bacillus* sp. N16-5 wild-type strain; ∆*ldh*, *Bacillus* sp. N16-5∆*ldh* strain; ∆*ldh*-pDlac, *Bacillus* sp. N16-5∆*ldh* strain carrying pDlac expression vector

Values were represented as the average ± standard deviation of three independent experiments

^a^ l-lactate product

^b^ d-lactate product

### Reducing the medium viscosity by disrupting EPS biosynthesis

Similar to other alkaliphiles, *Bacillus* sp. N16-5 produces EPS when grow in a high-salt environment [13]. Thus, the medium becomes viscous after fermentation, which complicates the post-harvesting stage. Additionally, we thought the production of additional EPS may decrease lactate production. The *epsD* gene is the key gene responsible for EPS biosynthesis [22]. Thus, the *epsD* gene in the *Bacillus* sp. N16-5∆*ldh*-pDlac strain was knocked out to inhibit EPS production. The resultant strain was designated as the *Bacillus* sp. N16-5∆*ldh*∆*epsD*-pDlac strain, and its EPS concentration, growth, and d-lactate and byproduct production were compared to those of the aforementioned strains. The WT, ∆*ldh*, and ∆*ldh*-pDlac strains produced approximately 1.99 to 2.21 g/l of EPS, while the ∆*ldh*∆*epsD* and ∆*ldh*∆*epsD*-pDlac strains had lower EPS concentrations, ranging from 0.62–0.68 g/l (Table 2). Kranenburg et al. [22] demonstrated that disrupting the *epsD* gene in *Lactococcus lactis* inhibited EPS production. Moreover, the *epsD* gene product is a glycosyltransferase that links the first sugar of the repeating unit to a lipid carrier when it is expressed in *E. coli*. The ∆*ldh*∆*epsD*-pDlac strain still produced some EPS because *Bacillus* sp. N16-5 also contains at least six other genes that encode group 1 glycosyltransferases that function similarly to the *epsD* gene product. Thus, these genes may compensate for the lack of *epsD* gene activity. Moreover, it was not necessary to disrupt others glycosyltransferase because if it were disrupted, it may have some negative effect on cell growth. Example, one of the genes is encoding dihydrodipicolinate reductase which is an enzyme that plays a role in lysine biosynthesis. However, the *epsD* gene appears to play an important role in EPS production in *Bacillus* sp. N16-5. Although the ∆l*dh*∆*epsD*-pDlac strain still produced some EPS, deleting the *epsD* gene was sufficient to reduce the viscosity of the medium after fermentation. When observed the viscosity with Brookfield viscometer, the culture viscosity was significantly reduced from 25.84 ± 1.44 to 5.84 ± 1.44 mPa s when compared with the WT strain. Using this strain enabled the clarification of the culture supernatant via a one-step centrifugation procedure after fermentation in Horikoshi as well as peanut meal-based media (data not shown).

**Table 2:** EPS, lactate, and byproduct formation in *Bacillus* sp. N16-5 wild-type and engineered strains

Strain	EPS (g/l)	Pyruvate (g/l)	Succinate (g/l)	Lactate (g/l)	Formate (g/l)	Acetate (g/l)
WT	1.99 ± 0.13	0.36 ± 0.01	0.71 ± 0.01	3.64 ± 0.00^a^	0.33 ± 0.00	3.74 ± 0.04
∆*ldh*	2.21 ± 0.03	2.86 ± 0.20	1.21 ± 0.02	0.64 ± 0.06^a^	0.40 ± 0.04	3.65 ± 0.16
∆*ldh*∆*epsD*	0.68 ± 0.03	2.88 ± 0.09	1.19 ± 0.02	0.77 ± 0.01^a^	0.34 ± 0.02	3.56 ± 0.01
∆*ldh*-pDlac	2.09 ± 0.16	0.34 ± 0.01	0.46 ± 0.02	5.68 ± 0.23^b^	0.12 ± 0.02	2.95 ± 0.12
∆*ldh*∆*epsD*-pDlac	0.62 ± 0.13	0.35 ± 0.02	0.52 ± 0.04	4.93 ± 0.22^b^	0.07 ± 0.01	3.62 ± 0.08

Values were represented as the average ± standard deviation of three independent experiments

*WT* wild-type

^a^ l-lactate product

^b^ d-lactate product

The single deletion strain and the double deletion strain did not differ in d-lactate production in preliminary batch fermentations (Table 3). This result implies that deleting the *epsD* gene did not influence d-lactate production as we expected. However, the double deletion strain is more suitable than the single deletion strain, as the ∆*ldh*∆*epsD*-pDlac strain reached a higher cell density. As a result, it produced more d-lactate in a shorter fermentation (Table 3). Thus, the ∆*ldh*∆*epsD*-pDlac strain is more favorable for d-lactate production because of its higher productivity and the simplicity of separating cells from the supernatant at the end of the fermentation process.

**Table 3:** d-Lactate production and cell growth in the single and double knocked out strains

Strains	Glucose consume (g/l)	D-lactate (g/l)	Yield (%)	Productivity (g/l/h)	Fermentation time (h)	OD_600_
∆*ldh*-pDlac	78.75 ± 0.08	60.13 ± 1.08	80.73 ± 0.01	2.86 ± 0.05	21.0	4.63 ± 0.02
∆*ldh*∆*epsD*-pDlac	78.75 ± 0.00	60.02 ± 1.42	80.56 ± 0.02	3.08 ± 0.07	19.5	5.08 ± 0.37

Values were represented as the average ± standard deviation of two independent experiments

### Fermentation medium optimization

**Fig. 2:**
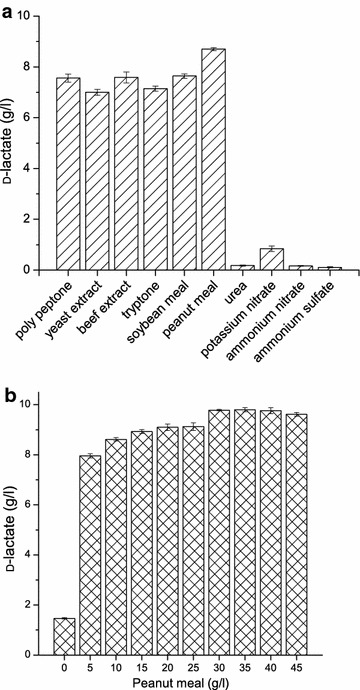
d-lactate production capacities of the engineered *Bacillus* sp. N16-5 strain using different nitrogen sources. **a** d-lactate production using different organic or inorganic nitrogen sources. **b** d-lactate production using different concentrations of peanut meal as a nitrogen source. *Error bars* represent the standard deviations of three replicates

To conduct a low-cost fermentation, various sources of organic or inorganic nitrogen were chosen to test their capacity for lactate production. The highest lactate production was obtained using peanut meal as a nitrogen source (Fig. 2a). Then, the concentration of peanut meal was further optimized. The d-lactate titers increased proportionally to the peanut meal concentrations from 0 to 30 g/l of peanut meal, while D-lactate production did not increase at peanut meal concentrations greater than 30 g/l (Fig. 2b). A nearly ideal yield of 100 % was achieved at a peanut meal concentration of 30 g/l.

After optimizing the peanut meal concentration, the effects of different salts on lactate production were also investigated. As shown in Table 4, only the addition of sodium acetate significantly promoted lactate production, and 2 g/l of sodium acetate yielded the highest lactate concentration. Lino et al. [23] reported that adding sodium acetate to the medium improved the growth and promoted lactic acid dehydrogenase activity, as well as lactic acid production, in *Lactobacillus* species. Our results demonstrate that the addition of sodium acetate also stimulated the growth and lactate production of alkaliphilic *Bacillus* strains.

**Table 4:** d-lactate production by the engineered strain when supplemented the medium with different kinds of salts

MnSO_4_•H_2_O (g/l)	D-lactate (g/l)	MgSO_4_•7H_2_O (g/l)	D-lactate (g/l)	K_2_HPO_4_•3H_2_O (g/l)	d-lactate (g/l)	CH_3_COONa (g/l)	D-lactate (g/l)
0.00	8.73 ± 0.03	0.00	8.91 ± 0.02	0.00	8.70 ± 0.05	0.00	9.44 ± 0.00
0.01	8.61 ± 0.00	0.10	8.78 ± 0.08	0.50	8.50 ± 0.02	0.50	9.37 ± 0.06
0.02	8.42 ± 0.06	0.20	8.81 ± 0.11	1.00	8.34 ± 0.10	1.00	9.44 ± 0.02
0.03	8.53 ± 0.05	0.30	8.59 ± 0.12	1.50	8.32 ± 0.07	1.50	9.36 ± 0.03
0.04	8.53 ± 0.01	0.40	8.63 ± 0.05	2.00	8.22 ± 0.08	2.00	9.49 ± 0.04
0.05	8.52 ± 0.03	0.50	8.72 ± 0.04	2.50	8.18 ± 0.01	2.50	9.37 ± 0.05
				3.00	7.90 ± 0.03	3.00	9.22 ± 0.05
						3.50	9.16 ± 0.02

Values were represented as the average ± standard deviation of three independent experiments

### Optimization of the fermentation conditions

Multiple batch fermentations were conducted to determine the optimal fermentation conditions for lactate production. The initial glucose concentration was first optimized in a batch fermentation by varying the concentration from 50 to 150 g/l. An initial glucose concentration of 80 g/l gave the highest yield of approximately 82.64 ± 0.01 % and the highest productivity of approximately 3.07 ± 0.03 g/l/h. Additionally, an initial glucose concentration of 80 g/l resulted in the best growth, as evidenced by the highest optical density at 600 nm (OD_600_) value (Table 5). Thus, an initial glucose concentration of 80 g/l was used in subsequent experiments. The optimum pH for the fermentation was determined by setting the pH to 8.5, 9.0, 9.5, or 10.0. Although a pH of 10.0 is optimal for *Bacillus* sp. N16-5 growth, a pH of 9.0 was chosen as the optimal pH for lactate production, as this resulted in the fastest lactic acid production rate (productivity) and yield (Table 6).

**Table 5:** d-Lactate production and growth of the engineered strain at different initial glucose concentrations

Initial glucose (g/l)	Glucose consume (g/l)	d-lactate (g/l)	Yield (%)	Productivity (g/l/h)	Maximum OD_600_
50	50.18 ± 0.01	31.51 ± 1.14	64.63 ± 0.02	2.25 ± 0.08	4.48 ± 0.20
80	72.49 ± 0.12	56.85 ± 0.64	82.64 ± 0.01	3.07 ± 0.03	5.30 ± 0.17
100	89.64 ± 0.15	67.32 ± 1.96	79.92 ± 0.02	2.49 ± 0.07	5.00 ± 0.27
120	109.35 ± 0.14	81.07 ± 1.64	79.94 ± 0.02	2.32 ± 0.05	4.78 ± 0.23
150	134.69 ± 0.03	96.46 ± 2.02	78.40 ± 0.02	1.68 ± 0.04	4.73 ± 0.48

Values were represented as the average ± standard deviation of two independent experiments

**Table 6:** Effects of pH on d-lactate production and growth of the engineered strain in batch fermentation

pH	Glucose consume (g/l)	d-lactate (g/l)	Yield (%)	Productivity (g/l/h)	Maximum OD_600_
8.5	74.44 ± 0.29	52.28 ± 1.10	73.68 ± 0.01	2.49 ± 0.02	4.15 ± 0.11
9.0	73.31 ± 0.07	55.24 ± 1.06	79.30 ± 0.01	2.91 ± 0.06	4.96 ± 0.25
9.5	72.84 ± 0.14	54.73 ± 1.17	79.02 ± 0.02	2.43 ± 0.05	4.13 ± 0.06
10.0	72.35 ± 0.16	43.33 ± 1.34	62.31 ± 0.02	1.52 ± 0.05	3.94 ± 0.17

Values were represented as the average ± standard deviation of two independent experiments

### Fed-batch fermentation under sterile and non-sterile conditions

**Fig. 3:**
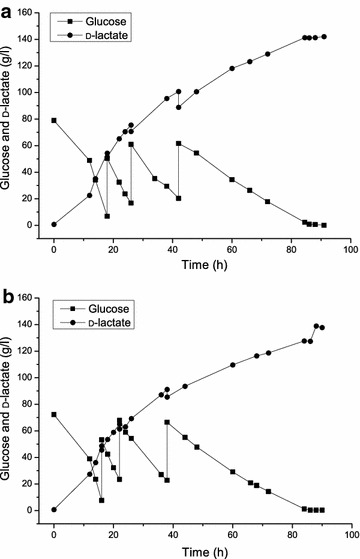
Fed-batch fermentation of d-lactate by the *Bacillus* sp. N16-5 ∆*ldh*∆*epsD*-pDlac strain. **a** A sterile fed-batch fermentations conditions and **b** a non-sterile fed-batch fermentations conditions

After the aforementioned optimizations, a medium containing 80 g/l glucose, 30 g/l peanut meal, and 2 g/l sodium acetate was used to perform a fed-batch fermentation. The fed-batch fermentation was conducted at 37 °C, with 100 rpm of agitation, and a static pH of 9.0, which was controlled by automatic feeding of 10 M NaOH. Two-stage aeration strategies were performed to reduce the length of the lag phase. A 1.0 lpm aeration was applied during the first 12 h of fermentation, and then aeration was stopped until the fermentation finished. The fed-batch fermentation was first conducted under sterile conditions. During the first 12 h of fermentation, glucose was slightly consumed and d-lactic acid production increased slightly. When aeration stopped, d-lactic acid production rapidly increased. This resulted in the highest productivity of approximately 3.02 g/l/h at 16 h. The final d-lactic acid concentration reached 142.05 g/l, with a yield of 94.25 % (Fig. 3a).

A non-sterile fermentation strategy may provide an opportunity to avoid the degradation of nutritional elements, which occurs during sterilization, during preparation of lactic acid fermentation [11]. A non-sterile fermentation process will simplify the fermentation process by reducing the number steps, the need for instruments, and operating costs, as well as by avoiding nutrient degradation via the Maillard reaction [24]. Thus, a non-sterile, fed-batch fermentation was conducted under the same conditions as those of the aforementioned sterile fermentation. Under non-sterile conditions, the d-lactic acid concentration reached 143.99 g/l, and a slightly higher yield of 96.09 % was achieved when compared with that of the sterile fermentation process (Fig. 3b). Moreover, the optical purity of d-lactic acid was 99.85 % under sterile and non-sterile conditions, which meets the requirements of the lactic acid polymerization process.

Because alkaliphilic organisms are considered to be potential organic acid producers [13], many scientists have attempted to find suitable alkaliphilic microorganisms for lactate production. To date, very few alkaliphiles have been reported to efficiently produce lactate. Calabia et al. [14] reported l-lactate fermentation by an alkaliphilic marine microorganism, which produced 59.6 g/l of lactic acid from 80 g/l glucose, with a yield of 76 % and an optical purity of 98.3 %. Yokaryo and Tokiwa [25] isolated several alkali-tolerant and alkaliphilic bacteria that produced lactic acid in an alkali broth. Among these, *Enterococcus casseliflavus* strain 79w3 produced a high concentration (103 g/l) of l-lactic acid, with a yield of 79.8 % and an optical purity of 99.5 %, during a batch fermentation. In our previous work, a very high l-lactate concentration of 225 g/l was achieved from a multi-pulse, fed-batch fermentation process by the alkaliphilic *Bacillus* sp. WL-S20 strain [9]. Additionally, the advantage of using alkaliphiles for lactate production is that it avoids contamination with neutrophilic microorganisms during fermentation under high pH conditions. Jiang et al. [15] reported that a 100 % optical purity of L-lactate was achieved by using an alkaliphilic *Exiguobacterium* sp. under non-sterile fermentation conditions. Notably, reducing the risk of contamination by DL-lactate producers is more important for d-lactate production. Until now, unlike l-lactate production by some thermotolerant *Bacillus* strains [7, 8], d-lactate fermentation was conducted at 37–42 °C, which increases the risk of contamination and lowers the optical purity. Alkaliphiles favor fermentation under high pH and salinity conditions; thus, their use could ensure the production of high-optical-purity d-lactic acid under mesophilic conditions.

To date, d-lactic acid production by alkaliphiles has not been reported. We first demonstrated that an engineered *Bacillus* sp. ∆*ldh*∆*epsD*-pDlac strain produced high-optical-purity d-lactate (99.85 %), and that this strain had several advantages, as NaOH was used as a neutralizer, peanut meal served as a low-cost nitrogen source, and the fermentation was conducted under non-sterile conditions. A high d-lactate concentration of 143.99 g/l, with a yield of 96.09 %, was achieved. Although Wang et al. [10] reported the highest D-lactate titer (>207 g/L), with an optical purity of 99.3 %, using calcium carbonate as a neutralizing agent to maintain the pH, the resultant large amount of insoluble calcium sulfate waste during the purification process has serious, adverse environmental consequences. In this study, the green neutralizer NaOH was used to maintain the pH during fermentation. The higher optical purity (99.85 %) and the cost-effective, non-sterile fermentation process developed in this study have the potential to produce polymer-grade d-lactate in an industrial setting.

## Conclusions

The challenge to use agricultural waste as nitrogen source is a slow cell growth and a low productivity. We engineered high optical purity d-lactate production of the *Bacillus* sp. ∆*ldh* -pDlac strain. The higher productivity of d-lactate was obtained via higher cell density by consequently disrupted *epsD* gene. Our engineered alkaliphilic *Bacillus* sp. ∆*ldh*∆*epsD*-pDlac strain can produce high-optical-purity d-lactate at a high titer using low-priced peanut meal as a nitrogen source and a NaOH-based, non-sterile fermentation process. The use of this strain at an industrial scale is favorable because of its simple process and low cost. Moreover, the use of a NaOH-based fermentation process is environmentally friendly because it does not generate precipitated waste.

## Methods

### Bacterial strains and vectors

*Bacillus* sp. N16-5 was used as the host, and it was cultivated in modified Horikoshi medium containing (g/l): glucose, 10; yeast extract, 5; polypeptone, 5; MgSO_4_·7H_2_O, 0.2; K_2_HPO_4_·3H_2_O, 1.31; and NaCl, 20 [13]. The pH was adjusted to approximately 10.0 after autoclaving by adding sterilized 10 % (w/v) Na_2_CO_3_. The medium was also used for seed cultures. The temperature-sensitive suicide vector pNNB194 was used to knock out desired genes [26]. Plasmid pMK4 was used as an expression vector [27]. *Escherichiacoli* DH5α was used for cloning and plasmid maintenance, and the pMD18-T vector (TaKaRa, Shiga, Japan) was used to carry the genes.

### Investigation of the Na-lactate tolerance of *Bacillus* sp. N16-5

*Bacillus* sp. N16-5 was cultivated in Horikoshi medium [13] containing 20 g/l glucose and different concentrations of NaCl (2, 5, 10, 15, 20, and 25 % w/v). Sodium lactate tolerance was observed by replacing NaCl with equimolar sodium lactate in the above Horikoshi medium (3.83, 9.58, 19.17, 28.77, 38.35, and 47.94 % w/v, respectively). Bacteria were grown at 37 °C, and samples were taken every 2 h to measure cell growth, as determined by OD_600_.

### Knocking out the *L*-*ldh* gene and the *epsD* gene in *Bacillus* sp. N16-5

The genome of *Bacillus* sp. N16-5 has been completely sequenced in our laboratory. One gene was annotated as a typical L-lactate dehydrogenase gene (*L*-*ldh*) (GenBank accession number: KT946599). To disrupt the *L*-*ldh* gene, gene-specific primers KLN16F (5′–TATATAGAAAGGACGATGTAAATGAGTG–3′) and KLN16R (5′–TCTTATCTTATTTGCCTGATCAAATGCC–3′) were designed to polymerase chain reaction (PCR) amplify the *L*-*ldh* gene and its 5′- and 3′-flanking regions. The fragment was cloned into pMD18T for maintenance. The *L*-*ldh* gene was excised from the plasmid by digestion with *Psi*I (New England Biolabs, Ipswich, MA, USA), and then the vector was re-ligated and introduced into *E. coli* DH5α. The knockout fragment was sub-cloned into pNNB194 by digestion with *Bam*HI and *Sal*I (New England Biolabs), and the resultant suicide vector was named pNNB-∆*ldh*. This vector is an *E. coli*/*B. subtilis* shuttle vector that contains an ampicillin resistance gene (*bla*) and an erythromycin resistance gene (*ermC*) for selection in *E. coli* and *B. subtilis*, respectively. The suicide vector was transformed into *Bacillus* sp. N16-5 by a protoplast transformation technique as described previously [19] and selected on SA5 plates containing erythromycin (0.5 μg/ml) at 34 °C. The targeted gene deletion was constructed by allelic exchange selection using the temperature shift method [26] by briefly increasing the temperature to the non-permissive temperature of 45 °C and plating the bacteria on neutral complex medium (NCM) plates [28] containing 0.5 μg/ml erythromycin to select for the integration of the suicide plasmid into the bacterial chromosome. To select clones in which the *L*-*ldh* gene was deleted, the temperature was lowered to the permissive temperature of 37 °C, and sequential subcultures of the bacteria were plated on NCM plates. The knockout strain was selected by replicating plating colonies on NCM plates with and without erythromycin (0.5 μg/ml), and the genotype was confirmed by PCR and sequencing. The resultant knockout strain was designated *Bacillus* sp. N16-5∆*ldh*.

Subsequently, the *epsD* gene that is responsible for exopolysaccharide (EPS) biosynthesis (GenBank accession number: KT946600) was knocked out using the same method, except that the knockout fragment was obtained by fusion PCR. Briefly, an upstream fragment was PCR amplified using primers QCepsDupF (5′–CGGGGTACCTGTTGCAACTGCTGCCCATAAC–3′) and QCepsDupR (5′–CACGACTGCATGCAAAATTCAAGGAGCCTCCTTCTATGATG–3′), and a downstream fragment was amplified using primers QCepsDdownF (5′–CATCATAGAAGGAGGCTCCTTGAATTTTGCATGCAGTCGTG–3′) and QCepsDdownR (5′–CGCGGATCCATGGAAAGACGAAGGCATCACACC–3′). Then, the two elements were fused by the overlapping PCR method. The knockout fragment was subcloned into pNNB194, and the knockout vector was named pNNB-∆*epsD*. The ∆*epsD* strain was confirmed by PCR using primers epsdF (5′–CTGAAGTGGTTTATCATGCTGCAGC–3′) and epsdR (5′–CAATTTCATGTGTGACGTGATCTG–3′) and sequencing. The resultant double knockout strain was designated *Bacillus* sp. N16-5∆*ldh*∆*epsD*.

### Construction of alkaliphilic d-lactate producer

pMK4 was used to express the d-lactate dehydrogenase gene (*D*-*ldh*) from *Lactobacillus delbrueckii* (GenBank accession number: 149576) under the control of the *L*-*ldh* gene promoter of *Bacillus* sp. N16-5. The *L*-*ldh* promoter was PCR-amplified using primers 165PRF (5′–GGAATTCCATATGCTGATGGTAGGACGCTTGTAC–3′; underline is *Nde*I site) and PR-LDH (5′–CGTAAGCAAAAATTTTAGTCATGTTTA AACATCTACCTTTCC–3′). The *D*-*ldh* gene was PCR amplified using primers LDH-PR (5′–GGAAAGGTAGATGTTTAAACATGACTAAAATTTTTGCTTACG–3′) and 165LDHR (5′–CGCGGATCCTTAGCCAACCTTAACTGGAG–3′; the *Bam*HI site is underlined). The expression fragment was obtained by fusing the genes by overlapping PCR, and the product was digested with *Nde*I and *Bam*HI (New England Biolabs) and ligated into the same sites in the pMK4 vector. Then, the expression vector was transformed into the desired knockout strains. The clone that carried the expression vector was selected on NCM plates containing 2.5 μg/ml chloramphenicol.

### Fermentation medium optimization

To determine the best nitrogen source for lactate production, 5 g/l of various organic and inorganic nitrogen sources were tested in media containing 10 g/l of glucose. Nitrogen sources were directly added when preparing the media and then autoclaved. A cheap peanut meal which is organic nitrogen rich containing 45.6 ± 2.8 % of protein was also tested [29]. A medium containing peanut meal was added a 0.22-µm filtered neutral protease to a final concentration of 0.1 g/ml of medium, and the peanut meal was hydrolyzed at pH 7.0 at 45 °C for 8 h before inoculation. Different salts at different concentrations, such as MnSO_4_·H_2_O at 0.00–0.05 g/l, MgSO_4_·7H_2_O at 0.00–0.50 g/l, K_2_HPO_4_·3H_2_O at 0.00–3.00 g/l, and CH_3_COONa at 0.00–3.50 g/l, were also investigated for lactate production. The inoculum volume was 10 % (v/v), and the experiments were conducted in shaking flasks without adjusting the pH. Samples were taken after 24 h of incubation, and the concentration of d-lactic acid was determined.

To optimize the initial glucose concentration and the fermentation pH, batch fermentation experiments were conducted in a 1.5–l bioreactor with a working volume of 700 ml of medium. The initial glucose concentration and fermentation pH were varied. The inoculum volume was 10 % (v/v). Temperature and agitation were 37 °C and 100 rpm, respectively. pH was controlled by automatically adding 10 M NaOH.

### Fed-batch fermentation

Fed-batch fermentation was initiated at an initial glucose concentration of 80 g/l. A pulse-feeding strategy was used by several additions of 45 ml of 75 % glucose when the residual glucose concentration was less than 20 g/l. pH was controlled by automatically feeding 10 M NaOH as a neutralizing agent. For the non-sterile strategy, all equipment, media, as well as the glucose solution, were not sterilized, while the peanut meal was sterilized by autoclaving at 121 °C for 15 min, and hydrolyzed at pH 7.0 at 45 °C for 8 h. A sample was collected at each time point to measure the concentrations of residual glucose and organic acids.

### Analytical methods

The OD_600_ was measured by a SpectraMax 190 spectrophotometer (Molecular Devices, Sunnyvale, CA, USA). EPS was measured by a modified EPS precipitation method [30, 31]. To determine the concentrations of glucose and other organic acids, samples were centrifuged at 10,000×*g* for 5 min, and the supernatant was analyzed by a high performance liquid chromatography system (1200 series, Agilent Technologies, Santa Clara, USA) with an Aminex HPX-87H column (300 × 7.8 mm) (Bio-Rad, Hercules, CA, USA) at 65 °C, a reflective index detector at 40 °C, and a UV detector at 215 nm. A solution of 18 mM H_2_SO_4_ was used as the mobile phase at a flow rate of 0.5 ml/min. The optical purity of D-lactic acid was determined by high performance liquid chromatography with a chiral column (MCI GEL CRS15 W, Mitsubishi Chemical, Tokyo, Japan) at 25 °C and a UV detector (254 nm), and 2 mM CuSO_4_ at a flow rate of 0.5 ml/min was used as the mobile phase. The optical purity of d-lactic acid was defined as: d-lactic acid/(d-lactic acid + l-lactic acid) × 100 %. The yield (%) was defined as: d-lactic acid (g)/consumed glucose (g) × 100 %.

## Abbreviations

Ldh: lactate dehydrogenase
EPS: exopolysaccharide
WT: wild-type
OD_600_: the optical density at 600 nm
NCM: neutral complex medium
